# Elucidating shared biomarkers in gastroesophageal reflux disease and idiopathic pulmonary fibrosis: insights into novel therapeutic targets and the role of angelicae sinensis radix

**DOI:** 10.3389/fphar.2024.1348708

**Published:** 2024-02-13

**Authors:** Xuanyu Wu, Xiang Xiao, Hanyu Fang, Cuifang He, Hanyue Wang, Miao Wang, Peishu Lan, Fei Wang, Quanyu Du, Han Yang

**Affiliations:** ^1^ Hospital of Chengdu University of Traditional Chinese Medicine, School of Clinical Medicine, Chengdu University of Traditional Chinese Medicine, Chengdu, China; ^2^ Graduate School, Beijing University of Chinese Medicine, Beijing, China; ^3^ Department of Traditional Chinese Medicine for Pulmonary Diseases, Center of Respiratory Medicine, China-Japan Friendship Hospital, Beijing, China

**Keywords:** idiopathic pulmonary fibrosis, gastroesophageal reflux disease, angelicae sinensis radix, mendelian randomization, network-pharmacology

## Abstract

**Background:** The etiological underpinnings of gastroesophageal reflux disease (GERD) and idiopathic pulmonary fibrosis (IPF) remain elusive, coupled with a scarcity of effective therapeutic interventions for IPF. Angelicae sinensis radix (ASR, also named Danggui) is a Chinese herb with potential anti-fibrotic properties, that holds promise as a therapeutic agent for IPF.

**Objective:** This study seeks to elucidate the causal interplay and potential mechanisms underlying the coexistence of GERD and IPF. Furthermore, it aims to investigate the regulatory effect of ASR on this complex relationship.

**Methods:** A two-sample Mendelian randomization (TSMR) approach was employed to delineate the causal connection between gastroesophageal reflux disease and IPF, with Phennoscanner V2 employed to mitigate confounding factors. Utilizing single nucleotide polymorphism (SNPs) and publicly available microarray data, we analyzed potential targets and mechanisms related to IPF in GERD. Network pharmacology and molecular docking were employed to explore the targets and efficacy of ASR in treating GERD-related IPF. External datasets were subsequently utilized to identify potential diagnostic biomarkers for GERD-related IPF.

**Results:** The IVW analysis demonstrated a positive causal relationship between GERD and IPF (IVW: OR = 1.002, 95%CI: 1.001, 1.003; *p* < 0.001). Twenty-five shared differentially expressed genes (DEGs) were identified. GO functional analysis revealed enrichment in neural, cellular, and brain development processes, concentrated in chromosomes and plasma membranes, with protein binding and activation involvement. KEGG analysis unveiled enrichment in proteoglycan, ERBB, and neuroactive ligand-receptor interaction pathways in cancer. Protein-protein interaction (PPI) analysis identified seven hub genes. Network pharmacology analysis demonstrated that 104 components of ASR targeted five hub genes (PDE4B, DRD2, ERBB4, ESR1, GRM8), with molecular docking confirming their excellent binding efficiency. GRM8 and ESR1 emerged as potential diagnostic biomarkers for GERD-related IPF (ESR1: AUC_*GERD*_ = 0.762, AUC_*IPF*_ = 0.725; GRM8: AUC_*GERD*_ = 0.717, AUC_*IPF*_ = 0.908). GRM8 and ESR1 emerged as potential diagnostic biomarkers for GERD-related IPF, validated in external datasets.

**Conclusion:** This study establishes a causal link between GERD and IPF, identifying five key targets and two potential diagnostic biomarkers for GERD-related IPF. ASR exhibits intervention efficacy and favorable binding characteristics, positioning it as a promising candidate for treating GERD-related IPF. The potential regulatory mechanisms may involve cell responses to fibroblast growth factor stimulation and steroidal hormone-mediated signaling pathways.

## 1 Introduction

Pulmonary fibrosis (PF) represents a progressive lung ailment characterized by compromised alveolar tissue repair following injury, attributed to diverse internal and external stimuli. Its clinical manifestations encompass a persistent dry cough and progressively worsening dyspnea (Mathai and Schwartz, 2019). Idiopathic pulmonary fibrosis (IPF) is the most common type of PF, with a median survival time of 2–4 years (Ogawa et al., 2021). Given its intricate pathogenesis, irreversible disease trajectory, heightened mortality rates, and unfavorable prognosis, IPF assumes characteristics akin to a neoplastic disorder (Aggarwal et al., 2016; Ma et al., 2020). Current therapeutic strategies for IPF remain nascent, while clinically approved agents like Pirfenidone and Nintedanib offer a mitigating effect on lung function decline but fall short in halting disease progression, coupled with a plethora of adverse reactions (Ma et al., 2022). While lung transplantation is the sole curative recourse for IPF (George et al., 2019), its widespread application is curtailed by post-transplant rejection and a scarcity of donor resources.

Observational studies have unveiled gastroesophageal reflux disease (GERD) as an independent risk factor for IPF, with 90% of IPF patients exhibiting GERD and a twofold increase in the risk of GERD among IPF patients (Khor et al., 2022; Raghu et al., 2006; Baqir et al., 2021). Gastric contents, including bile and pepsin, have been identified in bronchoalveolar lavage fluid (BALF) of IPF patients (Davis et al., 2013; Lee et al., 2012; Lozo et al., 2014). Intriguingly, the infusion of gastric juice into the airway induces lung injury in animal models, evoking a local inflammatory response, abnormal myofibroblast activation, excessive deposition of extracellular matrix (ECM), and formation of PF (Davis et al., 2013; Hoppo et al., 2014). Accordingly, aggressive treatment of GERD will help prevent and improve the prognosis of IPF. However, current GERD intervention modalities remain constrained, with proton pump inhibitors offering symptomatic relief but falling short of achieving a definitive cure.

Traditional Chinese Medicine (TCM), with its millennia-long history, emerges as a pivotal avenue for disease prevention and treatment, offering a framework deeply rooted in TCM theory. Both clinical and preclinical studies underscore the positive impact of herbal medicine on intervening in IPF, contributing to enhanced life energy and improved blood circulation (Zhou et al., 2024). Angelicae Sinensis Radix (ASR), also named Danggui, a Chinese herbal medicine renowned for its blood-circulating and stasis-removing properties, has been demonstrated through modern pharmacology to mitigate inflammatory cell infiltration, reduce oxidative stress, restore the alveolar-capillary barrier, and ameliorate chronic pathological lung changes (Wei et al., 2016; Zhang et al., 2022; Guo et al., 2024). In addition, ASR has antifibrotic effects via regulation of inflammatory damage, epithelial-mesenchymal transition (EMT), ECM, and other pathways (Han et al., 2006; Gao et al., 2012; Qian et al., 2020; Zhang et al., 2020).

Despite advancements in utilizing Mendelian randomization (MR) to investigate the causal relationship between GERD and IPF (Reynolds et al., 2023; Cotton et al., 2023), existing studies have limitations, including incomplete confounding factor exclusion and exclusive focus on the causal link. Moreover, a comprehensive exploration of the genetic overlap, susceptibility gene sharing, and deep mechanisms between the two diseases is still lacking. To address these issues, this study explores the causal relationship between GERD and IPF utilizing MR and the possible internal targets and mechanisms of GERD-related IPF using single nucleotide polymorphisms (SNPs) combined with publicly available microarray data. Furthermore, the regulatory impact of ASR on GERD-related IPF is explored through network pharmacology and molecular docking, offering novel insights into ASR’s mechanism of action and presenting potential avenues for complementary and replacement anti-PF therapies.

## 2 Methods

### 2.1 Identification of genetic causality between GERD and IPF

#### 2.1.1 Study design

**FIGURE 1:**
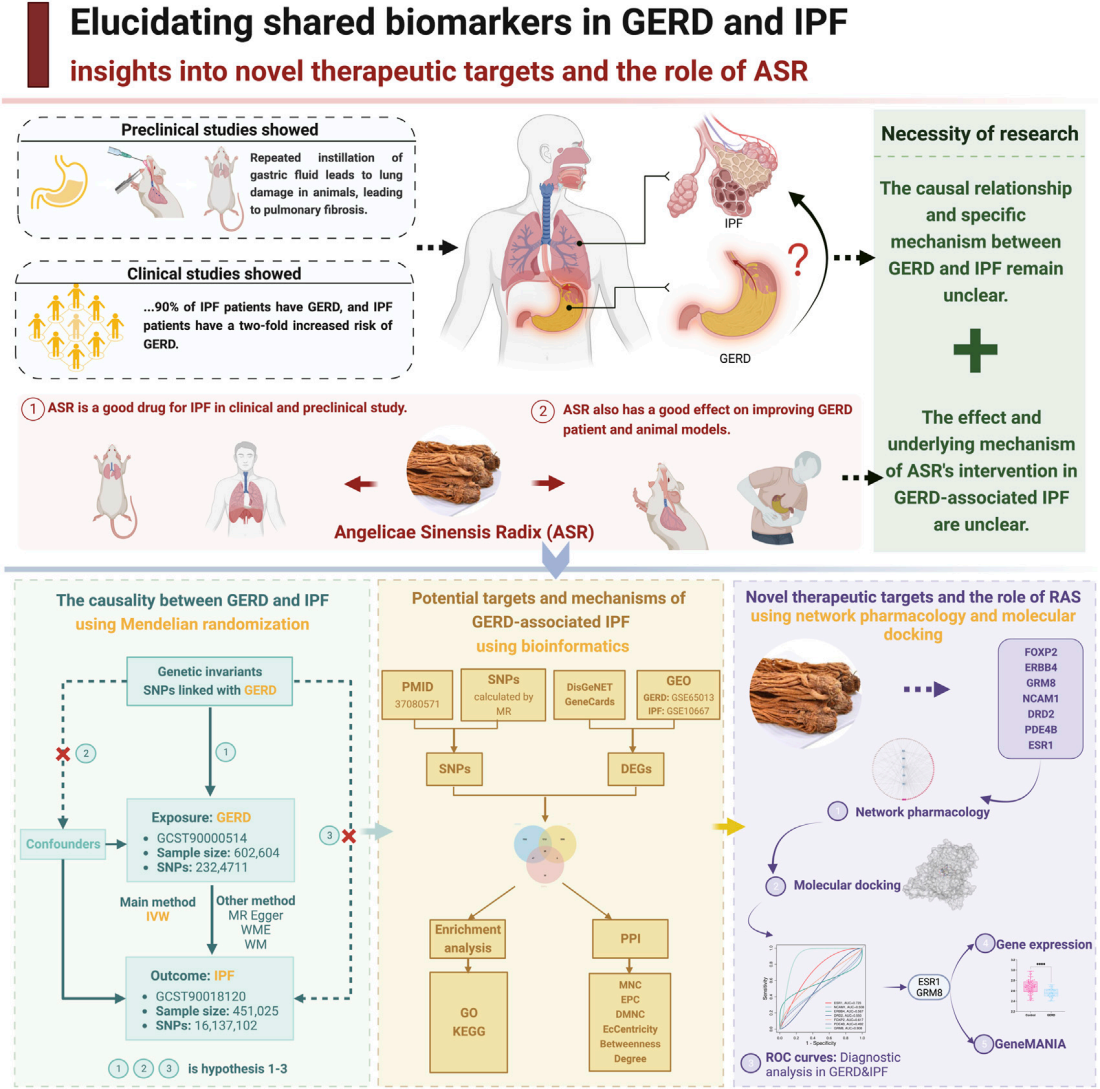
Study flowchart of the entire procedure. (Created by BioRender).

MR constitutes the foundation of this study, relying on genetic variants and employing the instrumental variable method. The methodological underpinning involves three core assumptions (Figure 1): Hypothesis 1: The genetic variant exhibits a robust association with the exposure (GERD). Hypothesis 2: The variant remains independent of any confounding factors. Hypothesis 3: Please check whether my interpretation.

#### 2.1.2 Data sources

In defining GERD as the exposure factor and IPF as the outcome factor, we assessed genome-wide association study (GWAS) summary data of GERD (GCST90000514) (Ong et al., 2022) and IPF (GCST90018120) (Duckworth et al., 2021) from the GWAS Catalog (https://www.ebi.ac.uk/gwas/). The GERD dataset comprised 602,604 samples, including 462,753 GERD patients and 1,127,474 controls, with genotyping information for 2,324,711 SNPs. The IPF dataset included 451,025 samples, with 1369 IPF patients, 435,866 controls, and genotyping details for 16,137,102 SNPs.

#### 2.1.3 Instrumental variables screening

Significant SNPs related to the exposure were selected based on a threshold of *p* < 5 × 10^−8^. We ensured SNPs independence by setting the linkage disequilibrium coefficient (r^2^ = 0.001) and region width (kb = 1,000) to eliminate linkage disequilibrium and prevent bias. IPF is associated with various risk factors, including genetics, autoimmune deficiency, environmental exposure (such as smoking, diabetes, GERD), and viral infection, among others (Reynolds et al., 2023; Zhao et al., 2023). Furthermore, GERD is associated with obesity, smoking, and genetics (Maret-Ouda et al., 2020). Recognizing the influence of confounders on both exposure and outcome (Zhou et al., 2023), we identified smoking and genetics as confounders. Utilizing the PhenoScanner database, we screened SNPs associated with confounders under conditions of *p* = 5 × 10^−8^, None Proxies, r^2^ = 0.8, and Build = 37. Weak instrumental variables were identified using *F* = $\left(N-2\right)\times \frac{R^{2}}{\left(1-R^{2}\right)}$ , where *R* ^*2*^ $=2\times \left(1-\text{EAF}\right)\times \text{EAF}\times {\left(\frac{\beta }{\text{SE}\times \sqrt{N}}\right)}^{2}$ , with *F* < 10 indicating the removal of weak instrumental variables. Palindromic SNPs with intermediate allele frequencies were excluded during the harmonization of exposure and outcome data.

#### 2.1.4 MR analysis

Inverse variance weighted (IVW) served as the primary MR analysis method, supplemented by weighted median (WM), simple mode (SM), MR-Egger regression, and weighted mode (WME) to assess IVW method robustness. Cochrane’s Q and leave-one-out (LOO) sensitivity tests evaluated the heterogeneity of individual genetic variation estimates. The IVW random effects model was applied in cases of heterogeneity among SNPs. Outliers were examined using MR-PRESSO, and horizontal pleiotropy was tested using MR-Egger-intercept.

#### 2.1.5 Statistical analysis

R (version 4.3.0) software packages “Two-Sample-MR” and “MR-PRESSO” facilitated all data analysis and visualization. Calculated results were expressed as odds ratios (OR) and 95% confidence intervals (95%CI), considering *p* < 0.05 as considered significant. Sensitivity analysis with *p* > 0.05 indicated no significant heterogeneity of SNPs, and MR-PRESSO verified the absence of outliers with *p* > 0.05. MR-Egger-intercept confirmed no horizontal pleiotropy with *p* > 0.05.

### 2.2 The screening of shared susceptibility gene between GERD and IPF

#### 2.2.1 Microarray data acquisition and processing

SNPs from MR calculations and literature, related to genes, were collated, removing duplicates and SNPs lacking corresponding gene names (Reynolds et al., 2023). Using “Gastroesophageal reflux disease” and “idiopathic pulmonary fibrosis” as search terms in the GEO database (https://www.ncbi.nlm.nih.gov/geo/), DisGeNET database (https://www.disgenet.org), and GeneCards database (www.genecards.org), respectively. Finally, GSE65013 (GERD) and GSE10667 (IPF) were selected for obtaining differentially expressed genes (DEGs) related to IPF-related GERD. Targets from DisGeNET and GeneCards were restricted to “*Homo sapiens*” and filtered based on the median score.

#### 2.2.2 Screening of shared DEGs between GERD and IPF

DEGs were analyzed using the limma package in R software, setting a threshold of “Adjusted *p* < 0.05 and log_2_(FC) > 1.5 or log_2_(FC) <-1.5". A heatmap was generated using the R package “pheatmap.” Shared genes of GERD-related IPF were identified through a Venn plot summarizing genes from multiple sources, including the GEO database, SNPs, DisGeNET, and GeneCards database.

#### 2.2.3 Functional analysis and protein-protein interaction (PPI) networks of shared DEGs

Metascape databases facilitated gene ontology (gene ontology, GO) and Kyoto Encyclopedia of Genes and Genomes (KEGG) enrichment analysis using shared DEGs (https://metascape.org/gp/index.html). Sangerbox (http://sangerbox.com/tool.html) was used for data visualization. The STRING database (https://cn.string-db.org/) was used to conduct a PPI network analysis, with a median confidence of 0.4. Cytoscape was subsequently used to visualize the results (Shannon et al., 2003). The Cytohubba plugin utilized six algorithms (MNC, EPC, DMNC, EcCentricity, Betweenness, and Degree) to identify top targets using six algorithms. A Venn plot aided in selecting potential targets.

### 2.3 Network pharmacology analysis of ASR intervention in GERD-related IPF

#### 2.3.1 Drug compound-target acquisition and screening

Active compounds of ASR were collected from the TCMSP database and literature, with chemical structures obtained from PubMed. Those not found in SDF format were transformed and drawn into the corresponding SMILES structures. Since the traditional use of ASR is an oral decoction, the screening parameters included oral availability and drug likeliness. Swiss target provided corresponding targets, and PPI analysis mapped the compound-target phase. The compound-target format was imported into Cytoscape for network construction, and the Degree algorithm sorted and visualized the network.

#### 2.3.2 Molecular docking of active components of ASR with hub genes

Molecular docking is widely used in drug screening and often used to predict the relationship between disease targets and drug structural ligands. The top three compounds with the highest degree value for each target were selected to screen compounds for molecular docking. Only the existing compounds were used for docking if the corresponding compounds were less than three. The 3D structures of small molecule compounds were downloaded from the PubChem database and stored in mol2 format. The 3D structures in the hub genes with a resolution lower than 2.5Å were downloaded from the PDB (https://www.rcsb.org/) database and saved in the PDB format. CB - Dock2 (https://cadd.labshare.cn/cb-dock2/), a tool that improves blind docking methods by integrating cavity detection, docking, and homology simulation fitting, was used to explore effective receptor-ligand binding sites (Liu et al., 2022a). The vina scores were finally used to calculate the binding energies between receptor-ligand molecules, with lower binding energies indicating stronger interactions. Binding energies < −5 kCal/mol represent favorable binding activity.

### 2.4 Identification and validation of diagnostic biomarkers for GERD-related IPF

Utilizing the pROC package in R, we generated ROC curves for key targets associated with GERD and IPF in GSE10667 and GSE39491 datasets. The diagnostic efficiency of each target for the two diseases was assessed by calculating the area under the curve (AUC). AUC values exceeding 0.7 indicated the identification of targets with good diagnostic efficiency. Subsequent screening and adjustment, considering the F value, removed six weak instrumental variables (rs1011407, rs11645288, rs2232423, rs3172494, rs3828917, rs569356). The GeneMANIA (http://www.genemania.org) database was then employed to predict potential interacting genes and pathways associated with the identified diagnostic biomarkers, offering preliminary insights into underlying mechanisms.

## 3 Results

### 3.1 MR analysis of the causal relationship between GERD and IPF

#### 3.1.1 Instrumental variables selection

After removing the linkage disequilibrium, 90 SNPs remained. PhenoScanner eliminated four SNPs related to tobacco intake (rs6711584, rs1510719, rs10242223, rs215614) and one SNPs related to gene expression (rs13107325). Subsequently, six weak instrumental variables (rs1011407, rs11645288, rs2232423, rs3172494, rs3828917, rs569356) were removed after screening and adjustment by calculating the *F* value. The harmonization process excluded three SNPs with intermediate allele frequencies, resulting in 76 instrumental variables.

#### 3.1.2 Positive causal effect of GERD on IPF

**FIGURE 2:**
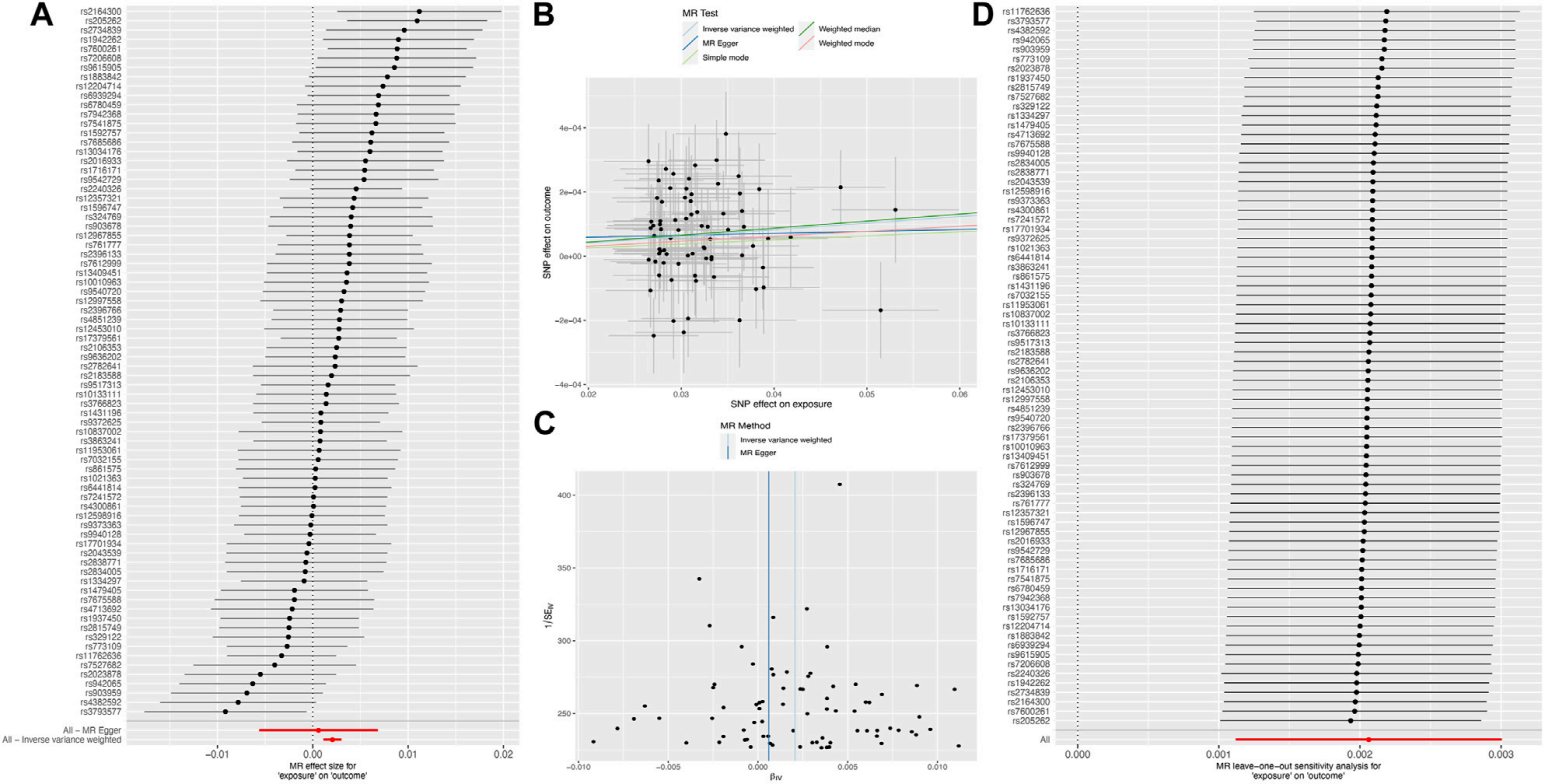
Results of MR **(A)** Forest plot; **(B)** Scatter plot; **(C)** Funnel plot; **(D)** Plot of LOO.

The MR analysis, employing the 76 instrumental variables (Table 1), demonstrated a positive causal relationship between GERD and IPF (IVW: OR = 1.002, 95% CI: 1.001, 1.003; *p* < 0.001; Figure 2A). Moreover, the scatterplot revealed that the results of WM, SM, MR-Egger regression, and weighted plurality method were consistent with IVW (Figure 2B), suggesting good robustness of the results. Cochrane’s Q test yielded *p* = 0.16, indicating no heterogeneity of SNPs within the study. The funnel plot showed that the distribution of causal effects was symmetrical, indicating no obvious bias (Figure 2C). The MR-Egger-intercept test yielded a *p*-value of 0.643, indicating the absence of pleiotropy, suggesting that the instrumental variables did not significantly affect the outcome through pathways other than exposure. The forest plot demonstrated that the MR analysis results were consistent upon excluding individual SNPs, as indicated by the LOO analysis (Figure 2D). MR-PRESSO test *p* = 0.207, no outlier SNPs were detected.

**TABLE 1:** Causal effect of GERD on IPF.

Exposure-outcome	Method	N (SNP)	*P*	OR	95%CI	Cochran’s Q test	MR-Egger-intercept	MR-PRESSO
GERD-IPF	MR Egger	76	0.850	1.001	0.994–1.007	0.163	0.643	0.207	
	WM	76	0.001	1.002	1.001–1.003				
	IVW	76	<0.001	1.002	1.001–1.003				
	SM	76	0.446	1.001	0.998–1.005				
	WME	76	0.372	1.002	0.998–1.005				

### 3.2 Shared susceptibility genes between GERD and IPF

#### 3.2.1 Common targets and shared DEGs between GERD and IPF

**FIGURE 3:**
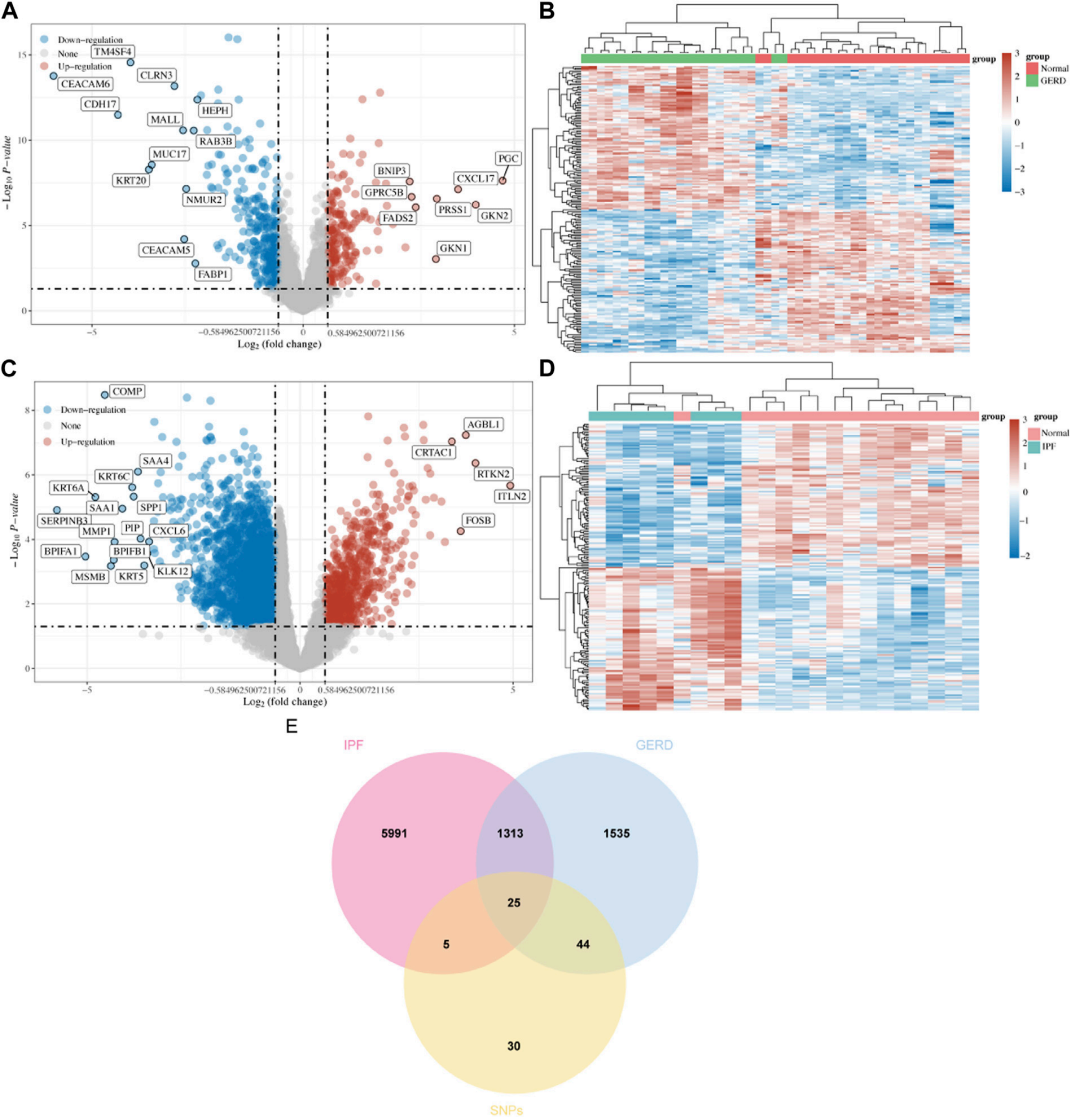
Screening potential targets for GERD-related IPF. **(A)** Volcano diagram revealed DEGs of GERD in GEO dataset; **(B)** Heat map revealed DEGs between GERD patients and healthy control group; **(C)** Volcano diagram revealed DEGs of IPF in GEO dataset; **(D)** Heat map revealed DEGs between IPF patients and healthy control group; **(E)**Venn plot revealed the intersection of shared DEGs between GERD and IPF.

Combining the 76 SNPs from MR analysis with those from the literature yielded 86 SNPs, and 104 corresponding genes were obtained from the GWAS catalog (Supplementary Table S1). The Limma package identified 522 DEGs for GERD (215 upregulated and 307 downregulated, Figures 3A, B), and 3,781 DEGs for IPF (734 upregulated and 3,047 downregulated, Figures 3C, D). From DisGeNET and GeneCards databases, 1532 GERD-related genes and 5994 IPF-related genes were obtained, respectively. The Venn plot showed 25 shared DEGs for GERD-related IPF (Figure 3E).

#### 3.2.2 Functional enrichment analysis

**FIGURE 4:**
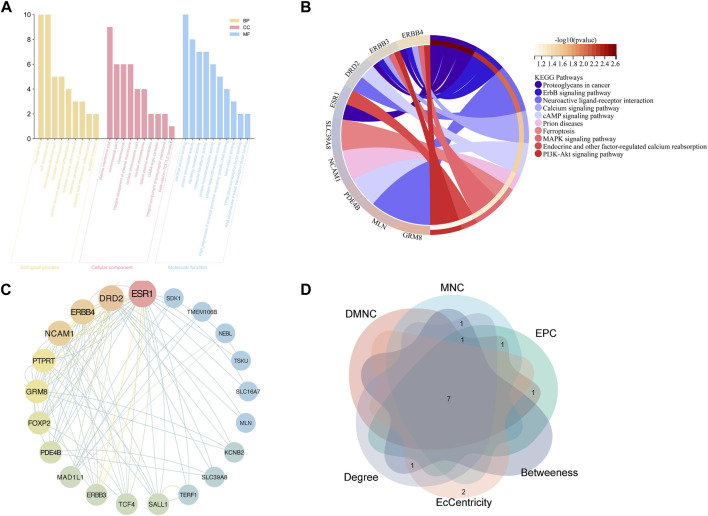
Functional enrichment analysis and PPI analysis of potential targets of GERD-related IPF **(A)** GO analysis; **(B)** KEGG analysis; **(C)** PPI network: potential genes; **(D)** Venn plot: top ten genes derived from six different algorithms.

GO functional analysis (Figure 4A) was employed to evaluate the enrichment of biological process (BP), cellular component (CC), and molecular function (MF), respectively. The study found that the BP terms were mainly enriched in neurogenesis, cell development, telencephalon, and forebrain development, whereas CC terms were mainly enriched in the plasma membrane, chromosomal part, chromosome, and integral component of the plasma membrane. MF terms were mainly enriched in protein binding, dimerization activity, signaling receptor activity, molecular transducer activity, and protein homodimerization activity. Based on the KEGG analysis, the potential targets were enriched in pathways, including proteoglycans in cancer, erythroblastic leukemia viral oncogene homolog, neuroactive ligand-receptor interactions, and calcium signaling pathway. These findings offer avenues for deeper exploration (Figure 4B).

#### 3.2.3 PPI network analysis

The STRING database facilitated PPI network analysis, and Cytoscape aided network visualization (Figure 4C). Top targets were identified using six algorithms, including MNC, EPC, DMNC, EcCentricity, Betweenness, and Degree, a Venn plot was drawn to visualize the intersection (Figure 4D), which contained seven hub genes, namely, Forkhead box p2 (FOXP2), Erb-B2 Receptor Tyrosine Kinase 4 (ERBB4), Glutamate Metabotropic Receptor 8 (GRM8), Neural Cell Adhesion Molecule 1 (NCAM1), Estrogen Receptor 1 (ESR1), Dopamine Receptor D2 (DRD2), Phosphodiesterase 4B (PDE4B).

### 3.3 Results of network pharmacology analysis of ASR intervention in GERD-related IPF

#### 3.3.1 Compounds-target acquisition and screening of ASR

**FIGURE 5:**
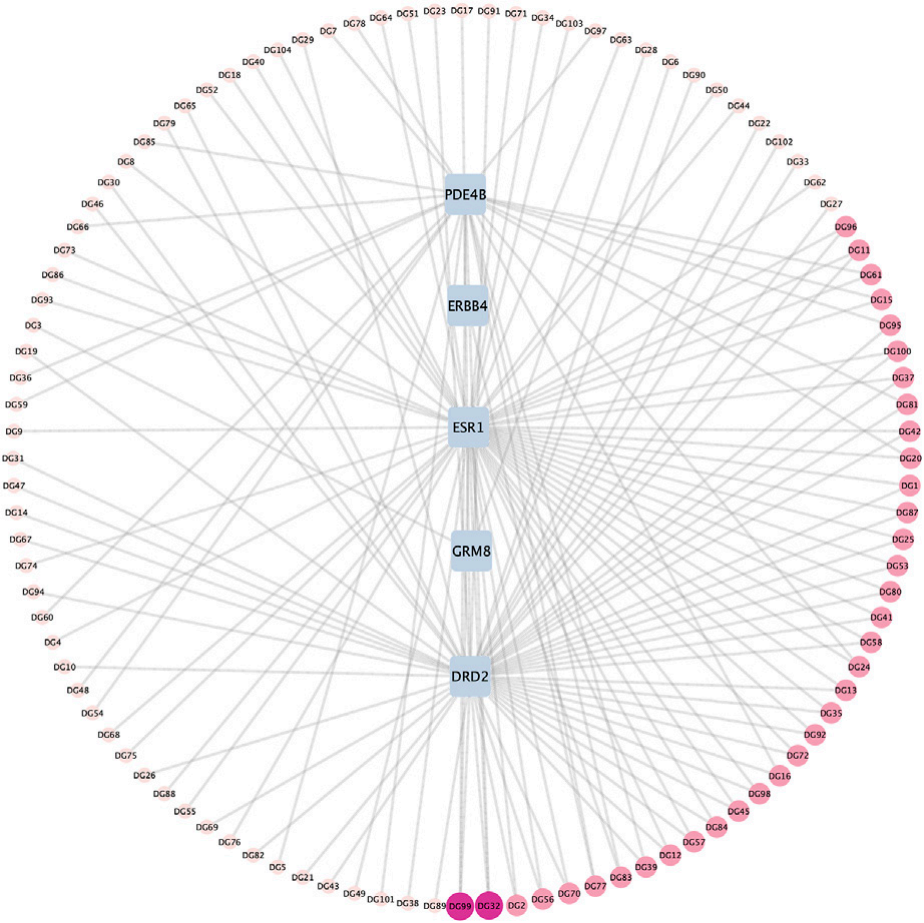
The network diagram of ASR compounds-target.

Utilizing TCMSP combined with a literature search (Feng et al., 2022), we amassed 360 active compounds of ASR. The small molecule structures of these compounds were retrieved from PubChem and subjected to oral availability and drug-likeness screening using SwissADME. Targets that could not be predicted in Swiss target were removed, resulting in a network of 109 nodes and 145 edges. Network pharmacology revealed that 104 ASR compounds had mapping relationships with five key targets (PDE4B, DRD2, ERBB4, ESR1, and GRM8) (Figure 5, Supplementary Table S2).

#### 3.3.2 Molecular docking verification of active compounds of ASR and hub genes of GERD-related IPF

**FIGURE 6:**
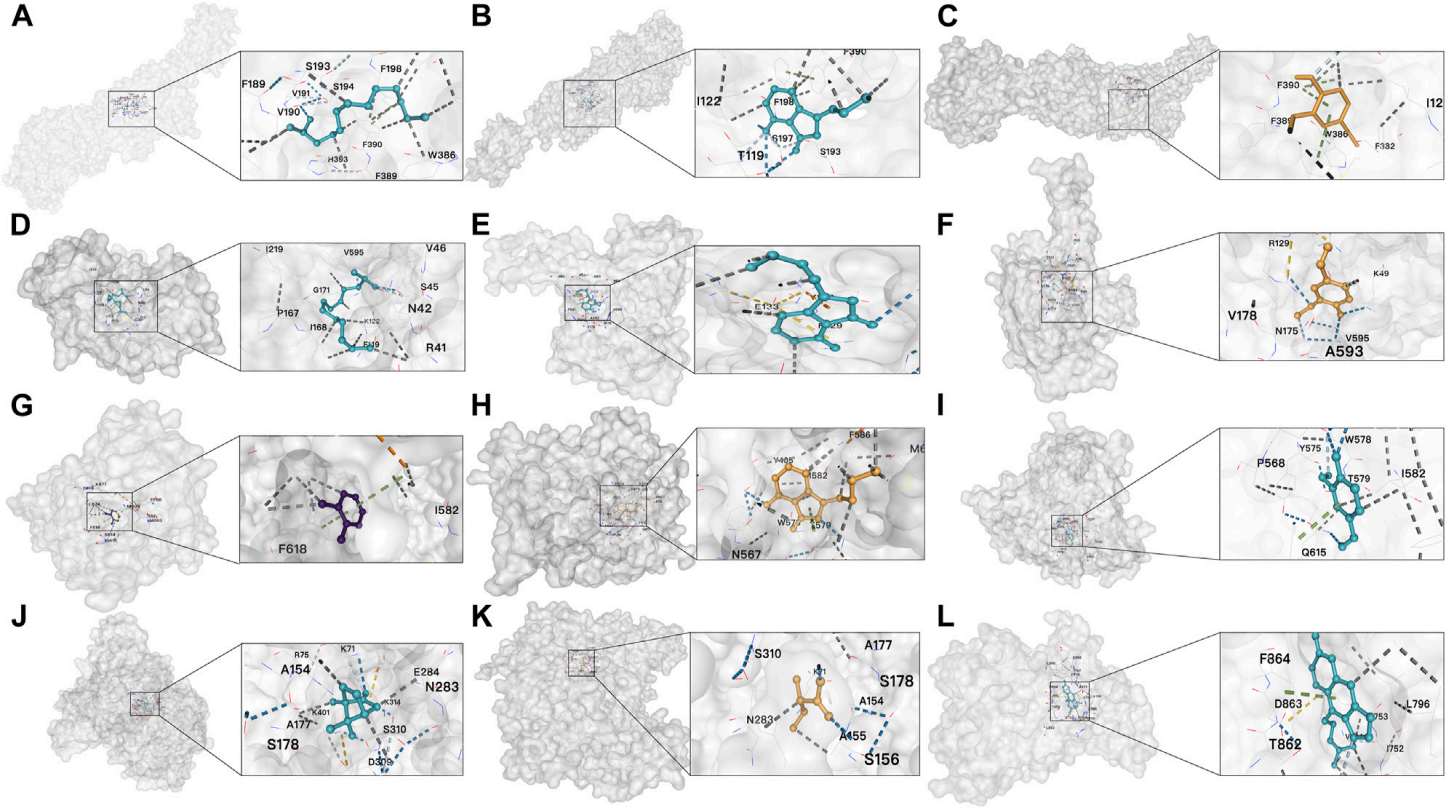
Results of molecular docking **(A)** Docking result between DRD2 and 2-TRIDECANOL; **(B)** Docking result between DRD2 and Senkyunolide B; **(C)** Docking result between DRD2 and Vanillin; **(D)** Docking result between ESR1 and 2-TRIDECANOL; **(E)** Docking result between ESR1 and Senkyunolide B; **(F)** Docking result between ESR1 and vanillin; **(G)** Docking result between PDE4B and o-cresol; **(H)** Docking result between PDE4B and Senkyunolide B; **(I)** Docking result between PDE4B and Vanillin; **(J)** Docking result between GRM8 and (±)-Camphor; **(K)** Docking result between GRM8 and 2-Methylbutyric acid; **(L)** Docking result between ERBB4 and Isoimperatorin.

The top three compounds corresponding to the five key targets were selected for molecular docking to assess the binding efficacy of ASR’s active compounds with disease targets. As there was only one corresponding active compound for ERBB4 and two for GRM8, molecular docking focused on these specific compounds. The results (Table 2) showed that the binding energies ranged from −4.5 to −8.2 kCal/mol, visually confirming excellent binding activity between ASR and the five key targets (Figure 6).

**TABLE 2:** Molecular docking results of five hub genes and ASR’s compounds.

Targets	PDB ID	Compounds	Cavity volume (Å^3^)	Affinity (kCal/mol)	Docking size			Center		
					X	Y	Z	X	Y	Z
ESR1	8APS	Vanillin	915	−5	17	24	25	−20	15	−10
		Senkyunolide B	915	5.9	19	19	25	−20	15	−10
		2-TRIDECANOL	915	4.5	25	25	25	−20	15	−10
DRD2	7DFP	Vanillin	1,338	5.4	17	17	26	−93	−22	214
		Senkyunolide B	1,338	7.8	19	19	26	−93	−22	214
		2-TRIDECANOL	1,338	5.9	25	25	25	−93	−22	214
PDE4B	4MYQ	Vanillin	3,802	−6	25	27	24	−16	38	−8
		Senkyunolide B	3,802	8.2	25	27	19	−16	38	−8
		o-cresol	3,802	5.8	25	27	24	−16	38	−8
GRM8	6BSZ	(±)-Camphor	1974	−6	16	24	27	−65	−21	34
		2-Methylbutyric acid	4,898	4.9	34	28	32	−44	−32	−23
ERBB4	7PCD	Isoimperatorin	3,541	7.9	26	28	34	4	−2	18

### 3.4 Screening of key targets and exploring the mechanism of GERD-related IPF

**FIGURE 7:**
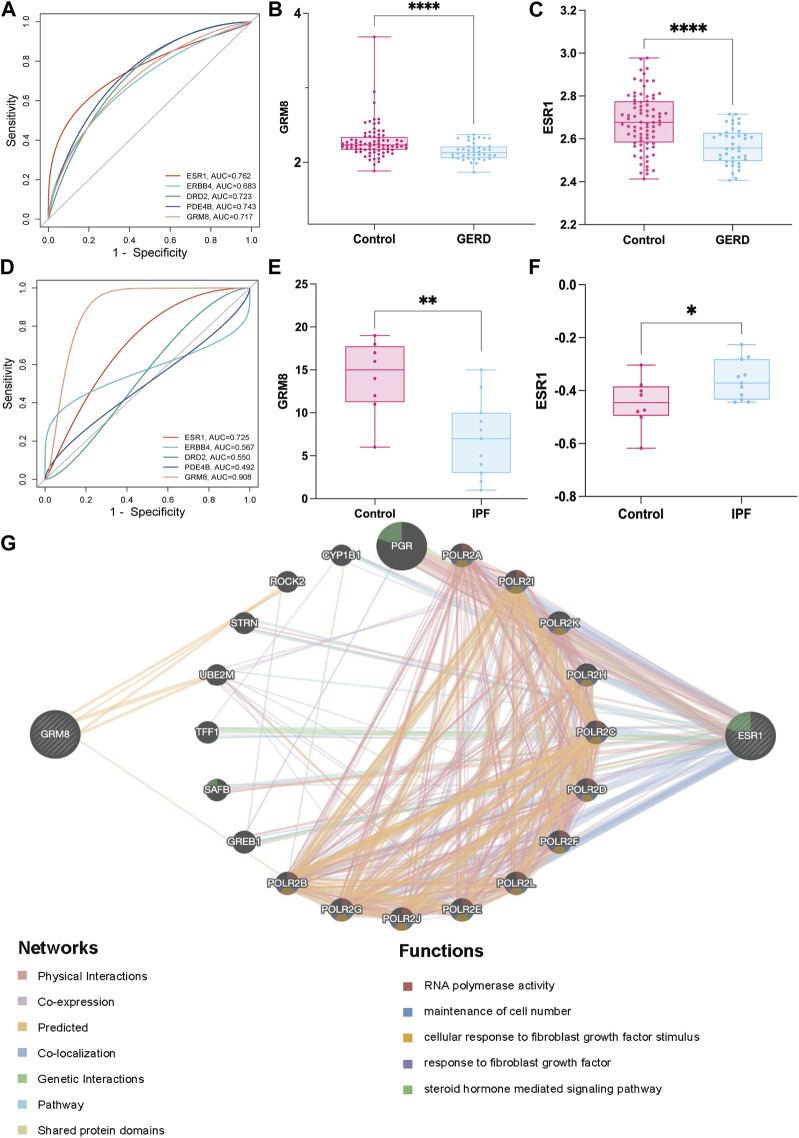
Screening and mechanism exploration of hub genes in GERD-related IPF **(A)** ROC curves of five targets in GERD; **(B)** Validation of GRM8 in GERD external dataset (GSE39491); **(C)** Validation of ESR1 in GERD external dataset (GSE39491); **(D)** ROC curves of the five targets in IPF; **(E)** Validation of GRM8 in IPF external dataset (GSE53845); **(F)** Validation of ESR1 in IPF external dataset (GSE53845); **(G)** Mechanisms exploration of two hub genes GRM8 and ESR1 in GeneMANIA. *p < 0.05, **p < 0.001, ****p < 0.0001.

ROC curves for the five key targets were generated using the R package pROC. Diagnostic efficacy in GERD and IPF was evaluated through AUC analysis (Figures 7A,D). AUC values were as follows: ESR1 (AUC_*GERD*_ = 0.762, AUC_*IPF*_ = 0.725), ERBB4 (AUC_*GERD*_ = 0.663, AUC_*IPF*_ = 0.567), DRD2 (AUC_*GERD*_ = 0.723, AUC_*IPF*_ = 0.550), PDE4B (AUC_*GERD*_ = 0.743, AUC_*IPF*_ = 0.492), GRM8 (AUC_*GERD*_ = 0.717, AUC_*IPF*_ = 0.908). Of these, ESR1 and GRM8 exhibited good diagnostic efficacy for GERD and IPF. External datasets validation confirmed significant downregulation of GRM8 and ESR1 in GERD patients (Figures 7B, C), and in IPF patients, GRM8 was significantly downregulated (*p* = 0.0005), and ESR1 was significantly upregulated (*p* = 0.0397) (Figures 7E, F). ASR intervention influenced the expression of these genes, as indicated by GeneMANIA predictions (Figure 7G), suggesting potential links to RNA-polymerase activity, maintenance of cell number, cellular response to fibroblast growth factor stimulus, response to fibroblast growth factor, and steroid hormone-mediated signaling pathways. RNA-polymerase activity, maintenance of cell number, cellular response to fibroblast growth factor stimulus, response to fibroblast growth factor, and steroid hormone-mediated signaling pathways.

## 4 Discussion

IPF, the prevailing manifestation of interstitial lung diseases, is burdened with a gloomy prognosis, boasting a median survival of 2–4 years after diagnosis. Clinical heterogeneity and a lack of symptom specificity often contribute to misdiagnosis or diagnostic delays (Lamas et al., 2011). In its advanced stages, IPF may progress to right heart failure coupled with respiratory failure, culminating in fatality (Sgalla et al., 2016). Timely identification and diagnosis are imperative. The pathogenesis of IPF involves unknown factors inducing repeated injury and abnormal inflammation of lung tissue, which induces crosstalk of epithelial cells, ECM, and adjacent interstitial tissue. Significant risk factors for this process include virus infection, smoke exposure, and chronic aspiration of gastric contents (Hewlett et al., 2018). A prevalent digestive tract ailment, exhibits a global incidence of 2.5%–51.2%, with a growing trend (Eusebi et al., 2018). Epidemiology has proved that GERD is an independent risk factor for IPF, which may affect more than 80% of IPF patients and promote the progression of fibrosis (Oldham and Collard, 2017). Animal studies have also confirmed that decreased gastric acid pH can aggravate lung injury (King and Nathan, 2013). Despite increased awareness of the GERD-IPF association, the molecular interplay between these conditions remains enigmatic. This study endeavors to elucidate the causal relationship between GERD and IPF, shedding light on regulatory molecules in GERD-related IPF.

At present, MR studies have substantiated a positive causal link between GERD and IPF (Reynolds et al., 2023; Cotton et al., 2023). However, one study, while utilizing PhenoScanner for confounders comparison, failed to eliminate relevant confounders (Reynolds et al., 2023). Another multivariate MR study established a connection between high BMI and the GERD-IPF causal relationship, which waned after adjusting BMI (Cotton et al., 2023). Therefore, this study fills a gap by conducting a two-sample MR analysis to demonstrate the genetic perspective of the causal relationship between GERD and IPF. Literature retrieval identified potential confounders, and PhenoScanner aided their removal. Calculation of the F-value guided the exclusion of five weak instrumental variables. IVW results (IVW: OR = 1.002, 95%CI: 1.001, 1.003) affirmed the GERD and IPF causal relationship, corroborated by the WM algorithm. Rigorous sensitivity analyses using Cochrane’s Q test and LOO underscored the reliability of the findings. MR-Egger intercept and MR-PRESSO tests revealed no horizontal pleiotropy or outliers, bolstering the article’s reliability.

Current studies suggest that GERD-related IPF may be due to abnormal anti-reflux barrier structure/function and local abnormal inflammation caused by the original gastric contents (gastric juice, bile, pepsin, gastric acid, chylous particles, et al.) reflux into the respiratory tract and lung (Warren et al., 2017). The BALF of patients with IPF contains gastric contents such as juice, bile, and pepsin, with esophageal sphincter relaxation due to decreased pressure (Davis et al., 2013; Lee et al., 2012; Lozo et al., 2014; Gavini et al., 2015; Soares et al., 2011). In acute IPF exacerbations, BALF has demonstrated gastrointestinal bacteria transplantation (Molyneaux et al., 2017). Continuous instillation of Hydrochloric acid (HCL) into the airway of mice can simulate gastric acid reflux and cause acute and chronic lung injury. In addition, it can also lead to the upregulation of fibrosis markers such as transforming growth factor β (TGF-β), hydroxyproline, and ECM-related proteins like elastin, tenascin C, and fibronectin. The severity of this abnormal state depends on the concentration of HCL, gender, and age of the mice (Marinova et al., 2019; Colunga Biancatelli et al., 2021; Solopov et al., 2021). It is possible that activation of shared signal pathways could be a mechanism for the conditions. Using duodenal ligation to simulate occult reflux and microaspiration, it can be found that the GERD combined with BLM-induced PF animal model has the same increased expression of extracellular-regulated kinases (ERK) and TGF-β1/Smad compared with the classical BLM animal model (Li, M., et al., 2021). The ERK1/2 signaling pathway is one of the potential shared pathways. The expression of ERK1/2 in the distal gastroesophageal squamous mucosa of patients with GERD is abnormal. The abnormal activation of ERK1/2 in IPF can regulate cell proliferation and fibroblast differentiation by up-regulating α-SMA and promoting the proliferation of type 1 collagen (Weng et al., 2019). The TGF-β/Smad pathway is another potential shared pathway. TGF-β is the most important cytokine in the development of IPF. Inhibiting the binding of TGF-β and Smad proteins has become a key strategy of anti-PF in recent years (Xue et al., 2020). Long-term exposure to GERD can cause the upregulation of TGF-β1, Smad-3, α-SMA, and type I collagen in the esophageal epithelium of mice, promoting the expression levels of ROS, NADPH oxidase-4, and malondialdehyde (Yisireyili et al., 2019). The precise mechanism of GERD-related IPF remains elusive. Since IPF lacks a cure, a profound understanding of its pathogenesis and identification of effective intervention targets is paramount. This study explores the mechanism between GERD and IPF, incorporating conventional databases (GEO, DisGeNET, and GeneCards) and SNPs from MR analysis, providing a genetic perspective on disease localization and intervention targets (Zhang et al., 2020; Zhang et al., 2023).

GO functional analysis revealed that disease targets of GERD-related IPF are mainly enriched in neural, cellular, and brain developmental processes, with chromosomal and plasma membrane fractions associated with protein binding and activation. This concurrence aligns seamlessly with our original genetic perspective, providing a comprehensive understanding of the intricate mechanisms underpinning these two diseases. The embryonic development mechanism proves that the digestive and respiratory systems have the same germ layer origin. Except for the nasal epithelium, the epithelium of the respiratory system is differentiated from the original endoderm of the digestive tract. Fibroblast growth factors (FGFs), pivotal in IPF, wield substantial influence during embryonic germ layer development. FGFs facilitate critical crosstalk connections among vital organs like the lung, brain, and intestine, orchestrating their growth spatially and temporally (El et al., 2017). FGFs also play an essential role in regulating lung homeostasis. For example, the FGFR2b ligand can inhibit lung fibrosis and promote tissue repair and regeneration (Dorry et al., 2020). GERD has been shown to increase the expression of FGFs and promote the differentiation of lung fibroblasts, mediate the upregulation of α-SMA, and activate RhoA/ROCK signaling to participate in IPF(Huang et al., 2010; Chiang et al., 2020). Additionally, GERD, arising from neurotrophin expression disorders and inflammation in the esophageal smooth muscle, triggers neuronal abnormalities and impaired esophageal motility (Chen et al., 2015). The relevance of abnormal neurotrophin signaling extends to IPF pathogenesis, where brain-derived neurotrophic factor contributes to the Twsit/snail signaling pathway, leading to abnormal EMT in IPF(Cherubini et al., 2017). Furthermore, both IPF and GERD correlate with the onset of nervous system diseases, including cognitive impairment and dementia, exhibiting overlapping pathway mechanisms (Ong et al., 2022; Adewuyi et al., 2022; Tudorache et al., 2019; Willander et al., 2011).

KEGG analysis showed that the disease targets of GERD-related IPF were mainly enriched in cancer’s proteoglycan, ERBB, and neuroactive ligand-receptor interaction pathways. The proteoglycan pathway in cancer is closely associated with abnormal glycosylation metabolism. The advanced glycation end products (AGE)/receptor of advanced glycation end products (RAGE) is an important pathway for abnormal glycosylation metabolism and is associated with aging-related diseases, including IPF (Nedić et al., 2013; Machahua et al., 2016). Long-term reflux in GERD induces local inflammatory responses, and an incomplete ADP-ribosylation-dependent DNA damage response occurs (Han and Zhang, 2022). A sensitive indicator of glycosylation - the lectins (UEA-1, DBA, HPA, and PNA) are also significantly reduced (Neumann et al., 2007). In contrast, the basement membrane of IPF lung epithelial cells highly expresses RAGE, which mediates and accelerates inflammation and fibrosis (Oczypok et al., 2017; Yamaguchi et al., 2017). The ERBB family of transmembrane receptor tyrosine kinases (RTKs) consists of the epidermal growth factor receptors EGFR (ERBB1), HER2 (ERBB2), HER3 (ERBB3) and HER4 (ERBB4). Cohort studies have demonstrated that GERD is a significant risk factor for EGFR-mutant lung cancer (Choi et al., 2019), and acid bile salt mimics GERD exposure and enhances ERBB2-mediated activation of immune cell/inflammatory pathways (Patankar et al., 2022). Pathological activation of ERBB/Yes-Associated Protein (YAP) in IPF patients’ airway epithelium regulates epithelial-driven mesenchymal transition (Stancil et al., 2021), and EGFR-targeting drugs have a particular effect on alleviating the inflammatory and fibrotic process of IPF (Wang et al., 2022). In addition, as an autonomic nervous organ, the lung contains certain sensory nerve fibers essential in regulating cardiopulmonary function and maintaining human health (Lee and Yu, 2014). Neural activity receptor-ligand facilitates signaling between lung fibroblasts and neurons, and the abnormal expression of its receptor-ligand-related genes, such as FPR1, BDKRB2, MCHR1, NMUR1, CNR2, P2RY14, and PTGER3, is observed in IPF lung fibroblasts (Nguyen et al., 2021). In addition, several studies (Lee et al., 2018) have found that lung fibroblasts can be transformed into neurons under certain conditions to participate in tissue repair. The differentiation-stimulating effect of GERD on lung-resident cells (Chiang et al., 2020) may be related to this mechanism.

As a critical TCM herb, ASR boasts many compounds and exhibits multifaceted effects on multiple targets (Hao et al., 2024). Widely utilized in clinical settings for the treatment of PF and GERD, ASR and its compounds have garnered attention in previous studies for their ability to mitigate and reverse IPF. The mechanisms involve reducing oxidative stress and inflammation, inhibiting EMT and myofibroblast activation, and improving lung function and related indicators in PF patients and rat models. Notably, ASR and its compounds have also demonstrated improvements and interventions in the gastroesophageal mucosa in animal subjects. This leads us to a logical inference: Can ASR effectively act upon and intervene in the key targets associated with GERD-related IPF? To answer this question, we embarked on a network pharmacology study, focusing on the seven hub genes (FOXP2, ERBB4, GRM8, NCAM1, ESR1, DRD2, PDE4B) of GERD and IPF.

The outcomes of the network pharmacology analysis unveiled that ASR exerts intervention effects on five hub genes (PDE4B, DRD2, ERBB4, ESR1, and GRM8). Notably, ESR1 and GRM8 exhibit good diagnostic efficacy for both GERD and IPF. PDE4B, a member of the PDE family, plays a crucial role in regulating intracellular Cyclic Adenosine Monophosphate (cAMP) concentration, engaging in anti-fibrotic signaling. cAMP antagonizes the pro-fibrotic signaling cascade and mediates an increase in the endogenous anti-fibrotic regulator prostaglandin E2 (Chambers, 2022). Targeted inhibition of PDE4B subtype is an important direction in the current drug development for IPF (Henderson et al., 2020; Hermann et al., 2022). BI 1015550, a targeted inhibitor of PDE4B, has been shown in clinical studies to improve patient’s forced vital capacity effectively and is expected to be a clinical candidate following Nintedanib and Pirfenidone (Chambers, 2022). PDE4B also affects gastritis-associated gastric cancer, and targeted inhibition of PDE4B can be involved in inhibiting the process of inflammation-associated gastric cancer (Xu et al., 2023). Dopamine receptors regulate gastrointestinal motility and gastric tone, and DRD2 receptor activation inhibits gastric tone and gastrointestinal motility (Bove et al., 2019) and stimulates pepsinogen production (Liu et al., 2022b). Moreover, dopamine receptors are related to regulating immune cell functions such as inflammation. DRD2 can inhibit the transcription of cytokines in macrophages and maximize the activation of YAP, thereby participating in the process of fibrosis (Qing et al., 2022). As one of the ERBB family genes, the role of ERBB4 in fibrosis of various organs has been emphasized (Vermeulen et al., 2017), and it has been considered a potential drug target for treating IPF (Higo et al., 2022). It is also implicated in gastric cancer mutations, apoptosis, and immune functions, although its role in GERD warrants further elucidation (Lucas et al., 2022).

ESR1, responsible for mediating hepatic metabolism, is a pivotal player in GERD, where bile acid, a constituent of gastric content, is integral. Abnormalities in estrogen genes, including ESR1, have been observed in conditions like eosinophilic esophagitis, influencing esophageal barrier function (Wheeler et al., 2019), caused by increased IL-13. Epidemiologic studies have shown (Wang et al., 2009) that men are more likely to develop IPF, with women having a lower prevalence and higher survival rates. An increasing number of studies also provide evidence for the involvement of estrogen in the development of IPF (Shim et al., 2013; Sathish et al., 2015). Estrogen E2 can regulate lung respiratory function and rhythm through the ESR1 receptor. After exogenous induction of bronchial epithelial cells with TGF-β1, ESR1 expression was significantly reduced (Smith et al., 2018). A significant downregulation of ESR1 expression was found in PF models exposed to multi-walled nanotubes (Smith et al., 2019) or silica (Brass et al., 2010). In the subsequent validation with external datasets, ESR1 expression was significantly lower in GERD, while ESR1 expression was significantly higher in IPF compared with the control group. The paradoxical phenomenon was also found in previous *in vitro* and *in vivo* studies (Gharaee-Kermani et al., 2005). Therefore, the specific mechanism of ESR1 in IPF, GERD, and GERD-related IPF remains to be further discovered.

GRM8, a metabotropic glutamate receptor, intricately regulates glutamate neurotransmission and has established associations with various central nervous system (CNS) disorders, including major depression, schizophrenia, and autism (Li et al., 2016). Despite a dearth of direct studies delineating GRM8’s role in both IPF and GERD, the neuromodulatory functions of GRM8 intersect with both conditions. Lung fibroblasts exhibit reverse transporter glutamate proteins linked to low glutamatergic levels in neuropsychiatric disorders (Huang et al., 2010). The antioxidant N-acetylcysteine, relevant to both PF and CNS neuropsychiatric disorders, demonstrates antifibrotic effects on lung fibroblasts. This aligns with GRM8’s position as a downstream player in glutamate metabolism (Raghu et al., 2021). GRM8’s involvement in central neurotransmission also extends to addiction-related behaviors where genetic variations impact consumer product intake, a significant GERD risk factor (Nilsson et al., 2004; Gast et al., 2013). The bidirectional regulation of the brain-gut axis, involving glutamate, may contribute to impaired gastrointestinal function and motility, thereby influencing GERD (Saegusa et al., 2011; Filpa et al., 2016). Although current research is limited, a more profound understanding of GRM8’s mechanisms in these diseases requires extensive exploration.

To further explore the possible relationship between GRM8 and ESR1 in the intervention of ASR on GERD-related IPF, we used GeneMANIA to predict similar genes and potential mechanisms. Notably, ESR1 exhibited a richer array of potentially similar genes compared to GRM8. The PGR and POLR2 families emerged as closely associated with ESR1. The predicted pathways encompass RNA-polymerase activity, maintenance of cell number, cellular response to fibroblast growth factor stimulus, response to fibroblast growth factor, and steroid hormone-mediated processes.

Progesterone receptor (PGR) and ESR1 are steroid receptors primarily expressed in women, with high prognostic value in various diseases (Berardi et al., 2016; Hertz et al., 2016; Kriegsmann et al., 2020), including fibrotic disorders. PGR and ESR1 work together in FGF-stimulated cell proliferation, differentiation, migration, and angiogenesis, a critical pathway in forming IPF (Ka et al., 2007). Therefore, we hypothesize that the mechanism between GERD-related IPF may linked to the above signaling pathway. Additionally, the role of steroids in this regard deserves more attention in future studies.

RNA polymerase II (POLR2) transcribes enzymes for protein gene coding and whole genome processes (Schier and Taatjes, 2020). It plays an essential role in both IPF and GERD. The POLR2 plays a crucial role in the abnormal repair of respiratory epithelial cells (Le et al., 2021). It also develops esophageal acidic microenvironment pH by binding to eotaxin-3 protein, an essential target of eosinophilic esophagitis (Zhang et al., 2012). When ASR targets GRM8 and ESR1 for GERD-related IPF, POLR2 may act as a common downstream molecule. It suggests potential for further study.

It is essential to acknowledge certain limitations in this study. Firstly, although MR enhances result reliability by mitigating confounding factors, the exclusive focus on a European population restricts the study’s generalizability. Secondly, our reliance on existing data from online databases might entail missing information, and future research should explore new, undocumented chemicals or targets. Moreover, the effects of ASR on hub genes expression in GERD-related IPF warrant thorough pharmacodynamic and functional verification. Lastly, further studies are imperative to elucidate proposed mechanisms, including the involvement of ESR1/PGR in the gender-specific effects of steroid hormones on the diseases.

## 5 Conclusion

In conclusion, this study is the first to explore the causal relationship between GERD and IPF and the mechanisms based on MR combined with other bioinformatics methods. We identified a positive causal relationship between GERD and IPF and the shared DEGs between GERD and IPF. Seven hub genes were finally selected. Five key targets were identified to be interfered with by ASR through network pharmacology and molecular docking. ESR1 and GRM8 had sound diagnostic effects in treating GERD-related IPF and are considered diagnostic biomarkers. The response of cells to FGFs stimulation and steroid hormone-mediated signaling pathway may be the potential mechanisms of ASR in GERD-related IPF.

## Data Availability

The original contributions presented in the study are included in the article/Supplementary Material, further inquiries can be directed to the corresponding authors.
