# Supplementary material for: Elucidating shared biomarkers in gastroesophageal reflux disease and idiopathic pulmonary fibrosis: insights into novel therapeutic targets and the role of angelicae sinensis radix
Source: Front Pharmacol. 2024 Feb 13;15:1348708. doi: 10.3389/fphar.2024.1348708 (PMC10897002; doi:10.3389/fphar.2024.1348708)
Supplement: Supplementary file 1 [file DataSheet1.DOCX]

Supplementary Material

# Supplementary Table 1 *86 SNPs in GERD-associated IPF*

| **SNP** | **Beta** | **se** | **P** | **Gene** |
| --- | --- | --- | --- | --- |
| rs10010963 | 3.54 e-03 | 0.004 | 4.23 e-01 | / |
| rs10133111 | 1.41 e-03 | 0.004 | 7.05 e-01 | TRAF3 |
| rs1021363 | 2.56 e-04 | 0.004 | 9.47 e-01 | SORCS3 |
| rs10837002 | 8.11 e-04 | 0.004 | 8.53 e-01 | LINC02759 |
| rs11762636 | 3.27 e-03 | 0.003 | 2.63 e-01 | MAD1L1, FTSJ2 |
| rs11953061 | 6.67 e-04 | 0.004 | 8.78 e-01 | RNU4-69P |
| rs12204714 | 7.37 e-03 | 0.004 | 7.72 e-02 | ESR1 |
| rs12357321 | 4.34 e-03 | 0.004 | 2.75 e-01 | MIR1915HG, SKIDA1, NEBL |
| rs12453010 | 2.75 e-03 | 0.004 | 4.92 e-01 | LINC01982, CA10 |
| rs12598916 | 9.23 e-05 | 0.004 | 9.81 e-01 | GNPATP |
| rs12967855 | 3.86 e-03 | 0.003 | 2.54 e-01 | CELF4 |
| rs12997558 | 3.03 e-03 | 0.004 | 4.86 e-01 | / |
| rs13034176 | 6.00 e-03 | 0.004 | 1.22 e-01 | / |
| rs1334297 | 9.09 e-04 | 0.003 | 7.88 e-01 | RNA5SP30, PCDH17 |
| rs13409451 | 3.59 e-03 | 0.004 | 4.02 e-01 | / |
| rs1431196 | 8.52 e-04 | 0.004 | 8.14 e-01 | DCC |
| rs1479405 | 1.92 e-03 | 0.004 | 6.26 e-01 | RERG |
| rs1592757 | 6.20 e-03 | 0.004 | 1.10 e-01 | NIHCOLE, RNU6-334P |
| rs1596747 | 4.19 e-03 | 0.004 | 2.60 e-01 | Intergenic |
| rs1716171 | 5.44 e-03 | 0.004 | 1.41 e-01 | MPHOS, PH9, C12orf65 |
| rs17379561 | 2.72 e-03 | 0.003 | 3.80 e-01 | DPYD |
| rs17701934 | 4.05 e-04 | 0.004 | 9.27 e-01 | / |
| rs1883842 | 7.83 e-03 | 0.004 | 6.17 e-02 | PTPRT |
| rs1937450 | 2.43 e-03 | 0.004 | 5.12 e-01 | PDE4B |
| rs1942262 | 8.99 e-03 | 0.004 | 2.60 e-02 | TCF4, RNA5SP459 |
| rs2016933 | 5.54 e-03 | 0.004 | 1.87 e-01 | / |
| rs2023878 | 5.50 e-03 | 0.004 | 1.75 e-01 | CRTC1, C19orf60 |
| rs2043539 | 6.09 e-04 | 0.004 | 8.87 e-01 | TMEM106B |
| rs205262 | 1.10 e-02 | 0.004 | 3.49 e-03 | ILRUN, UHRF1BP1 |
| rs2106353 | 2.50 e-03 | 0.004 | 5.06 e-01 | GRM8 |
| rs2164300 | 1.12 e-02 | 0.004 | 1.09 e-02 | RNU6-699P |
| rs2183588 | 1.97 e-03 | 0.004 | 6.39 e-01 | / |
| rs2240326 | 4.56 e-03 | 0.002 | 6.35 e-02 | / |
| rs2396133 | 3.84 e-03 | 0.004 | 3.31 e-01 | / |
| rs2396766 | 2.92 e-03 | 0.004 | 4.17 e-01 | FOXP2 |
| rs2734839 | 9.60 e-03 | 0.004 | 2.17 e-02 | DRD2, TTC12, ANKK1 |
| rs2782641 | 2.35 e-03 | 0.004 | 5.94 e-01 | / |
| rs2815749 | 2.49 e-03 | 0.004 | 5.04 e-01 | RPL31P12, NEGR1 |
| rs2834005 | 7.93 e-04 | 0.004 | 8.50 e-01 | / |
| rs2838771 | 7.32 e-04 | 0.004 | 8.65 e-01 | ADARB1 |
| rs324769 | 4.03 e-03 | 0.004 | 3.54 e-01 | SLC16A7 |
| rs329122 | 2.55 e-03 | 0.004 | 5.29 e-01 | JADE2 |
| rs3766823 | 1.39 e-03 | 0.004 | 7.21 e-01 | ADGRB2, COL16A1 |
| rs3793577 | 9.16 e-03 | 0.004 | 3.46 e-02 | ELAVL2 |
| rs3863241 | 7.64 e-04 | 0.004 | 8.30 e-01 | KCNB2, TERF1 |
| rs4300861 | 7.78 e-05 | 0.004 | 9.84 e-01 | RN7SL117P, RNA5SP87 |
| rs4382592 | 7.83 e-03 | 0.004 | 6.05 e-02 | MED27 |
| rs4713692 | 2.16 e-03 | 0.004 | 6.19 e-01 | MLN, LINC01016 |
| rs4851239 | 2.80 e-03 | 0.004 | 4.40 e-01 | AFF3, LYG1 |
| rs6441814 | 2.33 e-04 | 0.004 | 9.55 e-01 | ABHD5, RNU6-367P |
| rs6780459 | 6.88 e-03 | 0.004 | 1.14 e-01 | / |
| rs6939294 | 6.90 e-03 | 0.004 | 6.94 e-02 | / |
| rs7032155 | 5.67 e-04 | 0.004 | 8.94 e-01 | / |
| rs7206608 | 8.81 e-03 | 0.004 | 3.81 e-02 | CDH13 |
| rs7241572 | 8.00 e-05 | 0.004 | 9.84 e-01 | KCNG2 |
| rs7527682 | 3.99 e-03 | 0.004 | 3.59 e-01 | / |
| rs7541875 | 6.62 e-03 | 0.004 | 1.20 e-01 | / |
| rs7600261 | 8.85 e-03 | 0.004 | 1.71 e-02 | ERBB4 |
| rs7612999 | 3.84 e-03 | 0.004 | 3.84 e-01 | ARPP21 |
| rs761777 | 3.85 e-03 | 0.004 | 3.17 e-01 | ADGRA1, KNDC1 |
| rs7675588 | 1.93 e-03 | 0.004 | 6.52 e-01 | PCAT4 |
| rs7685686 | 6.08 e-03 | 0.004 | 1.48 e-01 | HTT |
| rs773109 | 2.69 e-03 | 0.003 | 4.03 e-01 | CDK2, SUOX, ERBB3, RAB5B |
| rs7942368 | 6.64 e-03 | 0.004 | 1.13 e-01 | TSKU, GUCY2EP |
| rs861575 | 2.97 e-04 | 0.004 | 9.44 e-01 | / |
| rs903678 | 3.97 e-03 | 0.004 | 3.67 e-01 | IPO9 |
| rs903959 | 6.91 e-03 | 0.004 | 8.85 e-02 | MIR1302-7, MROH5 |
| rs9372625 | 8.49 e-04 | 0.003 | 7.88 e-01 | MMS22L |
| rs9373363 | 2.07 e-04 | 0.004 | 9.60 e-01 | HIVEP2 |
| rs942065 | 6.30 e-03 | 0.004 | 1.08 e-01 | UNC79, COX8C |
| rs9517313 | 1.61 e-03 | 0.004 | 6.54 e-01 | STK24, FARP1 |
| rs9540720 | 3.28 e-03 | 0.004 | 4.51 e-01 | PCDH9 |
| rs9542729 | 5.38 e-03 | 0.004 | 1.76 e-01 | B3GLCT |
| rs9615905 | 8.57 e-03 | 0.004 | 4.18 e-02 | TAFA5, FAM19A5 |
| rs9636202 | 2.36 e-03 | 0.004 | 5.29 e-01 | PGPEP1, RN7SL513P |
| rs9940128 | 2.81 e-04 | 0.004 | 9.36 e-01 | FTO |
| rs1011407 | 0.019 | 0.005 | 4.97 e-05 | BCL11A |
| rs13107325 | 0.045 | 0.005 | 6.45 e-17 | SLC39A8 |
| rs920559 | 0.025 | 0.004 | 1.24 e-09 | FLJ20021 |
| rs1510719 | 0.012 | 0.003 | 6.89 e-05 | MAML3 |
| rs2145318 | 0.016 | 0.003 | 3.31 e-08 | HMGN4, BTN2A1, BTN2A2, BTN1A1, BTN3A3 |
| rs215614 | 0.014 | 0.003 | 2.17 e-06 | PDE1C |
| rs10242223 | 0.013 | 0.003 | 4.01 e-05 | SDK1 |
| rs10789931 | 0.018 | 0.005 | 1.22 e-04 | NCAM1 |
| rs957345 | 0.014 | 0.003 | 1.43 e-06 | YLPM1, PROX2, EIF2B2 |

# Supplementary Table 2 *Results of network pharmacology: compounds - targets - nodes*

| **Compounds** | **Nodes** | **Targets** |  | **Compounds** | **Nodes** | **Targets** |
| --- | --- | --- | --- | --- | --- | --- |
| 2-TETRADECANOL | DG1 | DRD2 |  | borneol | DG53 | DRD2 |
| 2-TETRADECANOL | DG1 | ESR1 |  | Brefeldin A | DG54 | PDE4B |
| 2-TRIDECANOL | DG2 | DRD2 |  | Caffeic acid | DG55 | ESR1 |
| 2-TRIDECANOL | DG2 | ESR1 |  | Carvacrol | DG56 | ESR1 |
| (±)-Camphor | DG3 | GRM8 |  | Carvacrol | DG56 | DRD2 |
| (Z)-6,7-epoxyligustilide | DG4 | ESR1 |  | Caryophyllene oxide | DG57 | DRD2 |
| 1-(2-AMINO-3,5-DIMETHYLPHENYL)-ETHANONE | DG5 | PDE4B |  | Caryophyllene oxide | DG57 | ESR1 |
| 1,2-DIMETHOXY-4-N-PROPYLBENZENE | DG6 | DRD2 |  | Decanal | DG58 | DRD2 |
| 10-angeloylbutylphthalid | DG7 | PDE4B |  | Decanal | DG58 | ESR1 |
| 10-Undecenal | DG8 | ESR1 |  | Decursin | DG59 | PDE4B |
| 11S,16R － Dihydroxyoctadeca － 9Z,17 －diene － 12, 14 － diyn － 1 － yl acetate | DG9 | ESR1 |  | Dibutyl phthalate | DG60 | PDE4B |
| 1-Decen-3-ol | DG10 | DRD2 |  | Dibutyl succinate | DG61 | PDE4B |
| 1-Dodecanol | DG11 | ESR1 |  | Dibutyl succinate | DG61 | ESR1 |
| 1-Dodecanol | DG11 | DRD2 |  | Dihydroxy-6'-methoxyacetophenone | DG62 | ESR1 |
| 1-Heptadecanol | DG12 | ESR1 |  | DI-TERT-BUTYL GLUTARATE | DG63 | ESR1 |
| 1-Heptadecanol | DG12 | DRD2 |  | E － Coniferin | DG64 | ESR1 |
| 1-Tetradecanol | DG13 | ESR1 |  | ethyl 3,4,7-trimethyl-2,6-octadienoate | DG65 | DRD2 |
| 1-Tetradecanol | DG13 | DRD2 |  | Ethyl propionate | DG66 | PDE4B |
| 2-(4-Methylphenyl)-1H-imidazole | DG14 | DRD2 |  | Eucarvon | DG67 | DRD2 |
| 2,3-dimethylphenol | DG15 | PDE4B |  | Eudesmol | DG68 | ESR1 |
| 2,3-dimethylphenol | DG15 | ESR1 |  | Eugenol | DG69 | DRD2 |
| 2,4,5-trimethylbenzaldehyde | DG16 | DRD2 |  | Fenchol | DG70 | ESR1 |
| 2,4,5-trimethylbenzaldehyde | DG16 | PDE4B |  | Fenchol | DG70 | DRD2 |
| 2,4,6－ Trimethylbenzaldehyde | DG17 | DRD2 |  | Ferulic aldehyde | DG71 | ESR1 |
| 2,4,6-Octatrienal, (E,E,E) | DG18 | ESR1 |  | Gallacetophenone | DG72 | DRD2 |
| 2,4-dimethylbenzaldehyde | DG19 | DRD2 |  | Gallacetophenone | DG72 | ESR1 |
| 2,4-dimethylphenol | DG20 | PDE4B |  | Gentisic acid | DG73 | ESR1 |
| 2,4-dimethylphenol | DG20 | ESR1 |  | Globulol | DG74 | ESR1 |
| 2,5-Dimethylbenzaldehyde | DG21 | DRD2 |  | Guaiacol | DG75 | ESR1 |
| 2,5-Octadien-4-one, 5,6,7-trimethyl-, (2E)- | DG22 | DRD2 |  | Isoeugenol | DG76 | ESR1 |
| 2,6-Dimethylbenzaldehyde | DG23 | DRD2 |  | Isoimperatorin | DG77 | ERBB4 |
| 2-Butanone, 3-phenyl- | DG24 | ESR1 |  | Isoimperatorin | DG77 | PDE4B |
| 2-Butanone, 3-phenyl- | DG24 | PDE4B |  | Isopropyl myristate | DG78 | PDE4B |
| 2-Ethyl-hexan-1-ol | DG25 | DRD2 |  | Isovaleraldehyde | DG79 | DRD2 |
| 2-Ethyl-hexan-1-ol | DG25 | ESR1 |  | laevo-pinocarveol | DG80 | DRD2 |
| 2-Isopropylbenzaldehyde | DG26 | DRD2 |  | laevo-pinocarveol | DG80 | ESR1 |
| 2-Methylbutanal | DG27 | DRD2 |  | Lauryl aldehyde | DG81 | DRD2 |
| 2-Methylbutyric acid | DG28 | GRM8 |  | Lauryl aldehyde | DG81 | PDE4B |
| 2-octen-1-al | DG29 | DRD2 |  | N － Butylbenzenesulfonamide | DG82 | DRD2 |
| 2-OCTYNAL | DG30 | DRD2 |  | o-cresol | DG83 | PDE4B |
| 3，4－ Dimethylbenzaldehyde | DG31 | DRD2 |  | o-cresol | DG83 | ESR1 |
| Senkyunolide B | DG32 | DRD2 |  | Octanal | DG84 | DRD2 |
| Senkyunolide B | DG32 | ESR1 |  | Octanal | DG84 | ESR1 |
| Senkyunolide B | DG32 | PDE4B |  | Oplopandiol | DG85 | PDE4B |
| 3-Hexanol,2,5-dimethyl- | DG33 | DRD2 |  | Paeonol | DG86 | ESR1 |
| 4 － Ethylresorcinol | DG34 | ESR1 |  | palustrol | DG87 | DRD2 |
| 4,10-Aromadendranediol | DG35 | DRD2 |  | palustrol | DG87 | ESR1 |
| 4,10-Aromadendranediol | DG35 | ESR1 |  | patchouli alcohol | DG88 | ESR1 |
| 4'-Ethylpropiophenone | DG36 | PDE4B |  | p-Dimethylaminobenzaldehyde | DG89 | DRD2 |
| 4-Hydroxy-3-methoxystyrene | DG37 | ESR1 |  | p-tolylmethanol | DG90 | DRD2 |
| 4-Hydroxy-3-methoxystyrene | DG37 | DRD2 |  | Safranal | DG91 | DRD2 |
| 4'-Hydroxyacetophenone | DG38 | ESR1 |  | Senkyunolide C | DG92 | DRD2 |
| 4-hydroxyphenyl ethanone | DG39 | DRD2 |  | Senkyunolide C | DG92 | ESR1 |
| 4-hydroxyphenyl ethanone | DG39 | PDE4B |  | Senkyunolide D | DG93 | ESR1 |
| 4-Methyl-5-decanol, threo + erythro | DG40 | ESR1 |  | Senkyunolide E | DG94 | DRD2 |
| 4-nonanol | DG41 | ESR1 |  | Senkyunolide F | DG95 | DRD2 |
| 4-nonanol | DG41 | DRD2 |  | Senkyunolide F | DG95 | PDE4B |
| 4-terpineol | DG42 | ESR1 |  | Spathulenol | DG96 | DRD2 |
| 4-terpineol | DG42 | DRD2 |  | Spathulenol | DG96 | ESR1 |
| 5-Nonanone | DG43 | DRD2 |  | Stearolic acid | DG97 | PDE4B |
| 6-UNDECANOL | DG44 | ESR1 |  | Tristin | DG98 | ESR1 |
| 7-TETRADECANOL | DG45 | DRD2 |  | Tristin | DG98 | DRD2 |
| 7-TETRADECANOL | DG45 | ESR1 |  | Vanillin | DG99 | ESR1 |
| 8-QuinolineMethanol | DG46 | DRD2 |  | Vanillin | DG99 | DRD2 |
| Amyl ketone | DG47 | DRD2 |  | Vanillin | DG99 | PDE4B |
| Angelicide | DG48 | PDE4B |  | Verbenone | DG100 | DRD2 |
| Anisic acid | DG49 | ESR1 |  | Verbenone | DG100 | ESR1 |
| Bergapten | DG50 | ESR1 |  | α-Bisabol | DG101 | PDE4B |
| bicyclogermacren | DG51 | ESR1 |  | α-campholenaldehyde | DG102 | DRD2 |
| Bis ( 2 － methylpropyl) phthalate | DG52 | ESR1 |  | β-Bisabolen | DG103 | ESR1 |
| borneol | DG53 | ESR1 |  | Phenol,2-methoxy-4-(1E)-1-propen-1-yl- | DG104 | ESR1 |
